# Both plasma and tumor tissue miR-146a high expression correlates with prolonged overall survival of surgical patients with intrahepatic cholangiocarcinoma

**DOI:** 10.1097/MD.0000000000008267

**Published:** 2017-11-03

**Authors:** Ri-Xin Zhang, Zhi Zheng, Kai Li, Xin-Hua Wu, Ling Zhu

**Affiliations:** Department of Hepatobiliary & Pancreatic Surgery, The Central Hospital of Wuhan, Tongji Medical College, Huazhong University of Science and Technology, Wuhan, China.

**Keywords:** intrahepatic cholangiocarcinoma, miR-146a/b, overall survival, prolonged, surgical patients

## Abstract

The purpose of this study was to investigate the association of tumor tissue and plasma miR-146a/b expressions with the clinicopathological properties and overall survival (OS) in surgical patients with intrahepatic cholangiocarcinomas (ICC).

Eighty-seven patients with ICC were enrolled. Tumor tissue and plasma sample were collected and miR-146a/b expressions were assessed by quantitative polymerase chain reaction (qPCR). The median follow-up duration was 31 months, and the last follow-up date was January 2017.

miR-146a (*P* < .001) and miR-146b (*P* = .006) expressions in tumor tissue were positively associated with that in plasma. Tissue miR-146a was negatively correlated with age (*P* = .036), poor differentiation (*P* = .020), N stage (*P* = .020), and TNM stage (*P* = .007), as well as ECOG performance (*P* = .008), whereas plasma miR-146a was inversely associated with N stage (*P* = .003), TNM stage (*P* = .003), and ECOG performance (*P* = .011). Moreover, tissue miR-146b was negatively correlated with gender (*P* = .043) and T stage (*P* = .047). Kaplan-Meier curves suggested that high expression of tissue miR-146a (*P* < .001) and plasma miR-146a (*P* = .029) were correlated with prolonged OS. Nevertheless, no association of miR-146b expression in tumor tissue (*P* = .187) and plasma (*P* = .336) with OS was discovered. Univariate analysis indicated that both tissue miR-146a (*P* < .001) and plasma miR-146a (*P* = .035) could predict better OS, whereas multivariate analysis revealed that only tissue miR-146a (*P* = .001) high expression was an independent factor for prolonged OS.

Both plasma and tissue miR-146a expression correlated with favorable OS, whereas only tissue miR-146a was an independent prognostic biomarker in surgical patients with ICC.

## 1 Introduction

Intrahepatic cholangiocarcinomas (ICC), with the second highest incidence of all liver cancer accounting for the proportion of 5% to 20%, is a critical threat imperiling people's health worldwide.^[1,2]^ In the last 2 decades, accumulating evidence revealed that the morbidity and mortality caused by ICC are increasing gradually over the world.^[3]^ Multiple elements are involved in the development and progression of ICC, among which the main risk factors consist of cholangiolithiasis, hepatitis B virus (HBV) infection, and *Clonorchis sinensis* infection, as well as primary sclerosing cholangitis (PSC).^[3–5]^ Despite great improvements of early diagnosis, optimal treatments (eg, surgery, chemotherapy, and radiotherapy), as well as patients’ care, the prognosis of ICC is still far from satisfaction, the 5-year survival rate of ICC patients with complete resection is only 20% to 40%.^[6,7]^

microRNAs (miRNAs), belonging to a great family of small endogenous non-coding RNAs, regulate target genes and protein expression via binding to the 3′-untranslated regions (3′-UTRs) directly.^[8]^ miRNAs contribute to multiple molecule activities, such as cell proliferation, differentiation, migration, and apoptosis.^[9,10]^ Accumulating evidence has defined miRNAs as biomarkers in various carcinomas, including colorectal cancer, gastric cancer, breast cancer, and lung cancer.^[11–14]^ microRNA-146 family (miR-146) consists of miR-146a and miR-146b, both of them have the similar sequences in the mature miRNAs excluding 2 bases toward the 3′-end.^[15]^ Dysregulated miR-146 expression has been described in a variety of malignant tumors, such as gastric cancer, breast cancer, or even hepatocellular cancer.^[16–18]^ However, few studies about the effects of miR-146 expression on the prognosis of ICC patients have been explored. Therefore, the purpose of this study was to investigate the association of tumor tissue and plasma miR-146 a/b expressions with the clinicopathological properties and overall survival (OS) in surgical patients with ICC.

## 2 Methods

### 2.1 Patients

Eighty-seven patients with ICC underwent surgery at Department of Hepatobiliary & Pancreatic Surgery in The Central Hospital of Wuhan, Tongji Medical College, Huazhong University of Science and Technology, from May 2012 to April 2014 were recruited in this prospective cohort study. The diagnosis of ICC was confirmed by clinical features, pathological determination, and/or radiology (computerized tomography [CT], magnetic resonance imaging [MRI], or endoscopic retrograde cholangiopancreatography [ERCP]). Patients with other tumors (solid or blood), inflammatory diseases, severe infection history, cognitive impairment, life expectancy <6 months were excluded from this study.

No patient received neo-adjuvant systemic therapy before the operation, and after the surgery, all the patients received adjuvant therapy with catheter radiofrequency ablation, or chemotherapy by gemcitabine plus cis-platinum, and/or radiotherapy.

This study was approved by Ethics Committee of The Central Hospital of Wuhan, Tongji Medical College, Huazhong University of Science and Technology; the written informed consents were obtained from all patients.

### 2.2 Clinicopathological features and follow-ups

Clinical and pathological properties of ICC patients at baseline were collected including age, gender, hepatitis B surface antigen (HBsAg) status, hepatic C virus (HCV) status, smoke, drink, differentiation, TNM stage, and ECOG performance status. The median follow-up duration was 31 months, and the last follow-up date was January 2017. OS was calculated from the date of surgery to patients’ death or last follow-up.

### 2.3 Sample collection and RNA extraction

Tumor tissue samples were obtained during the surgery. Blood samples were collected before the operation and plasma were separated. Total RNA was then extracted from the samples with TRIzol reagent (Invitrogen) according to the instructions of the manufacturer.

### 2.4 miR-146a/b determination by qPCR

Total RNA was extracted from tumor tissue and plasma samples by Trizol LS kit (TaKaRa, Japan), and RNA was subjected to reverse transcription by utilizing the PrimerScript Real-time reagent kit (TaKaRa, Japan). Subsequently, miR-146 a/b expressions were quantitated by SYBR Premix Ex TaqTM II (TaKaRa, Japan). The primer sequences (BGI, China) for the primer source were performed as follows: hsa-miR-146a-5p RT primer: 5′-CTCAACTGGTGTCGTGGAGTCGGCAATTCAGTTGAGAACCCATG-3′; hsa-miR-146a-5p Forward primer: 5′-ACACTCCAGCTGGGTGAGAACTGAATTCCATG-3′; hsa-miR-146a-5p Revert: 5′-TGTCGTGGAGTCGGCAATTC-3′; hsa-miR-146b-5p RT primer; 5′-ACACTCCAGCTGGGTGAGAACTGAATTCCATG-3′; hsa-miR-146b-5p Forward primer: 5′-ACACTCCAGCTGGGTGAGAACTGAATTCCATA-3′; hsa-miR-146b-5p Revert: 5′-TGTCGTGGAGTCGGCAATTC-3′. The expression levels of miR-146a/b were calculated by using the 2^-△△t^ method and U6 served as the internal reference.

### 2.5 Statistics

Statistics was carried out using SPSS 22.0 (IBM) and 2010 office software (Microsoft). Correlation of miR-146a/b expression in tissue and plasma sample, as well as miR-146a/b expression with clinicopathological features were determined by Spearman test. Kaplan-Meier curves and log-rank test were used to compare OS in different groups. Univariate and multivariate Cox regression were used to evaluate the baseline predictive factors for OS in ICC surgical patients. *P* <.05 was considered significant.

## 3 Results

### 3.1 Patients characteristics

The median age was 57.0 (53.0–65.0) years, male and female were 58 (67%) and 29 (33%) (Table 1). Thirty-two (37%) patients were HBsAg positive and 10 (11%) patients were HCV positive, and 27 (31%) patients were with smoke history and 40 (46%) patients were with drink history. Meanwhile, there were 53 (61%) patients with moderate-well differentiation, but 34 (39%) patients with poor differentiation. The number of patients in I/II TNM stage was 45 (52%), and in III/IV TNM stage was 42 (48%). Other clinicopathological characteristics of ICC patients are shown in Table 1.

**Table 1:**
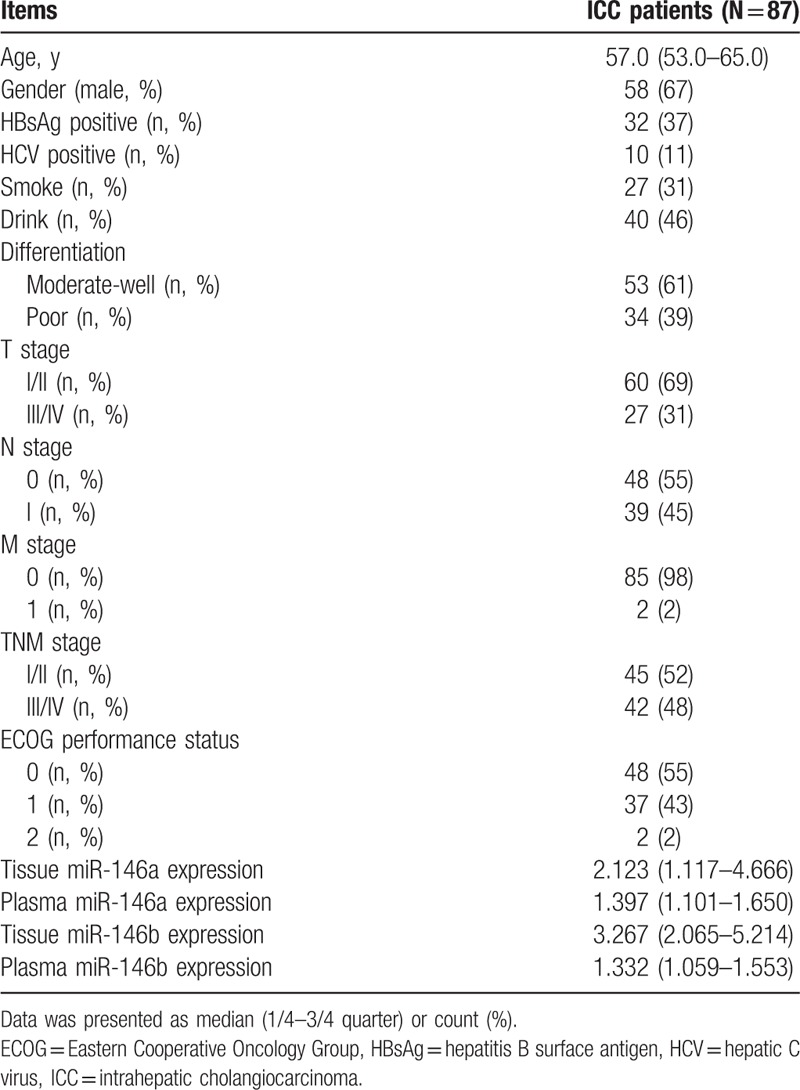
Baseline clinicopathological characteristics of ICC patients (N = 87).

### 3.2 Correlation between miR-146a/b in tumor tissue and plasma

**Figure 1:**
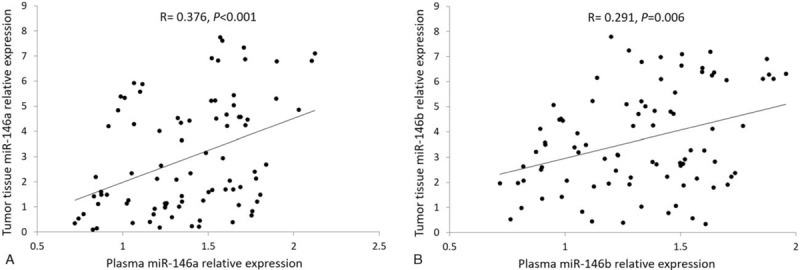
Correlation between miR-146a/b expressions in plasma and in tumor tissues. (A) Correlation between miR-146a expressions in plasma and in tumor tissues. (B) Correlation between miR-146b expressions in plasma and in tumor tissues. Spearman test was used to analyze the correlation of miR-146a/b expression in plasma with that in tumor tissue. *P* <.05 was considered significant.

Correlations of miR-146a/b in tumor tissue and plasma were evaluated in Figure 1. miR-146a (R = 0.376, *P* < .001, Fig. 1A) and miR-146b (R = 0.291, *P* = .006, Fig. 1B) expressions in tumor tissue were positively associated with that in plasma.

### 3.3 Correlation of miR-146a/b with clinical and pathological features

As listed in Table 1, miR-146a expressions in tissue and plasma samples were observed to be 2.123 (1.117–4.666) and 1.397 (1.101–1.650), respectively, whereas miR-146b expressions were 3.267 (2.065–5.214) in tissue samples, and 1.332 (1.059–1.553) in plasma samples. As presented in Table 2, tissue miR-146a was negatively correlated with age (R = −0.225, *P* = .036), poor differentiation (R = −0.250, *P* = .020), N stage (R = −0.249, *P* = .020), and TNM stage (R = −0.286, *P* = .007), as well as ECOG performance (R = −0.282, *P* = .008), whereas plasma miR-146a was inversely associated with N stage (R = −0.314, *P* = .003), TNM stage (R = −0.318, *P* = .003), and ECOG performance (R = −0.271, *P* = .011). Moreover, tissue miR-146b was negatively correlated with gender (R = −0.217, *P* = .043) and T stage (R = −0.214, *P* = .047), and plasma miR-146b was negatively associated with smoke (R = 0.228, *P* = .034).

**Table 2:**
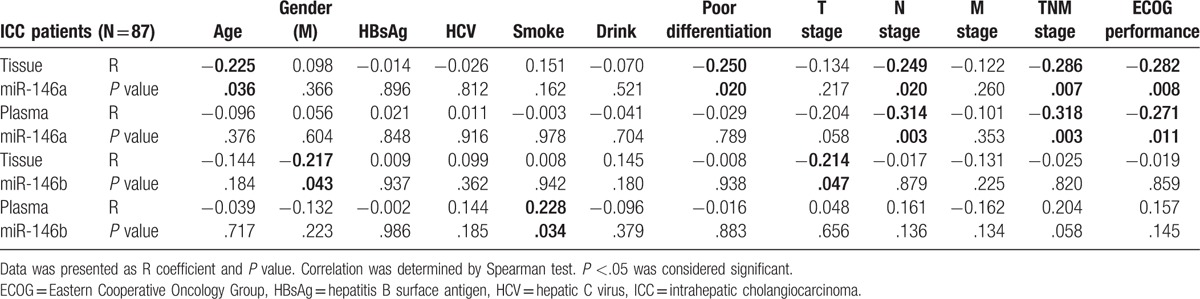
Correlation of miR-146a/b expression with clinicopathological features.

### 3.4 Tissue/plasma miR-146a correlated with prolonged OS but not miR-146b

**Figure 2:**
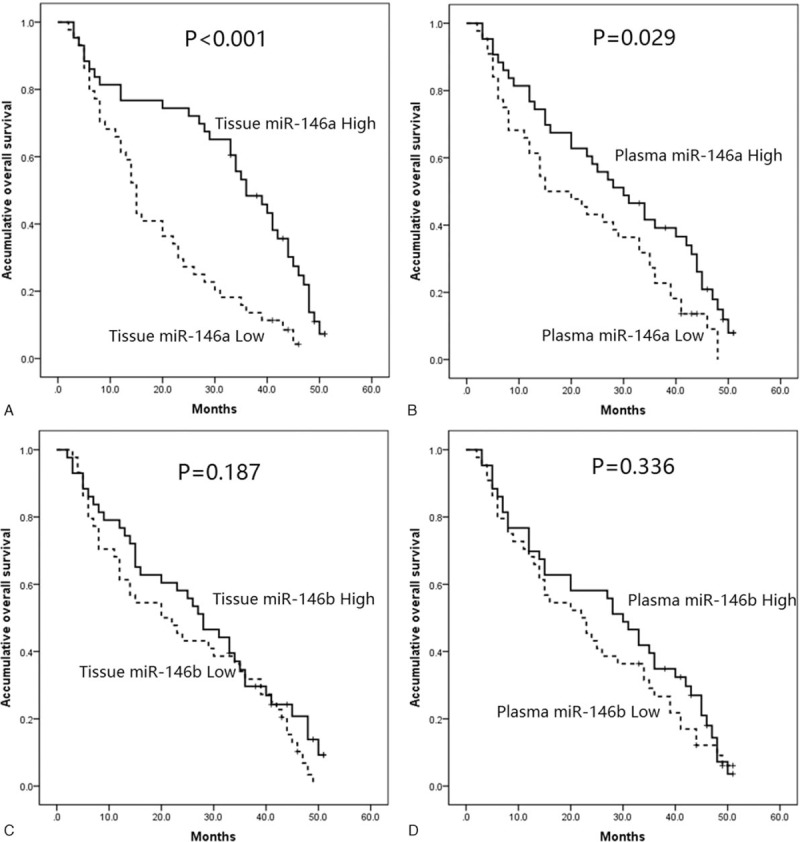
Association of plasma and tumor tissue miR-146a/b levels with OS. (A) Association of miR-146a with OS in tissue sample. (B) Association of miR-146a with OS in plasma sample. (C) Association of miR-146b with OS in tissue sample. (D) Association of miR-146b with OS in plasma sample. Kaplan-Meier curves and log-rank test were used to evaluate the correlation of plasma and tumor tissue miR-146a/b levels with OS. *P* <.05 was considered significant.

In terms of miR-146a, its high expression in both tumor tissue (*P* < .001, Fig. 2A) and plasma (*P* = .029, Fig. 2B) were correlated with prolonged OS compared with low expression. Nevertheless, no association of miR-146b expression in tumor tissue (*P* = .187, Fig. 2C) or plasma (*P* = .336, Fig. 2D) with OS was discovered in the present study.

### 3.5 Tissue miR-146a independently predicted longer OS

Univariate Cox proportional hazards regression was used to evaluate the baseline predictive factors for OS in ICC surgical patients, as presented in Table 3, which indicated that miR-146a high expression in tissue (*P* < .001) and in plasma (*P* = .035) were associated with better OS. Besides, poor differentiation (*P* = .045), higher T stage (III/IV) (*P* < .001), higher N stage (I) (*P* = .005), higher M stage (I) (*P* = .048), increased TNM stage (III/IV) (*P* = .001), and elevated ECOG performance status (1/2) (*P* = .007) were correlated with worse OS in surgical patients with ICC. Furthermore, ICC patients treated with catheter radiofrequency ablation compared with chemo(radio) therapy seemed to be associated with better OS, but without statistical significance (*P* = .234).

**Table 3:**
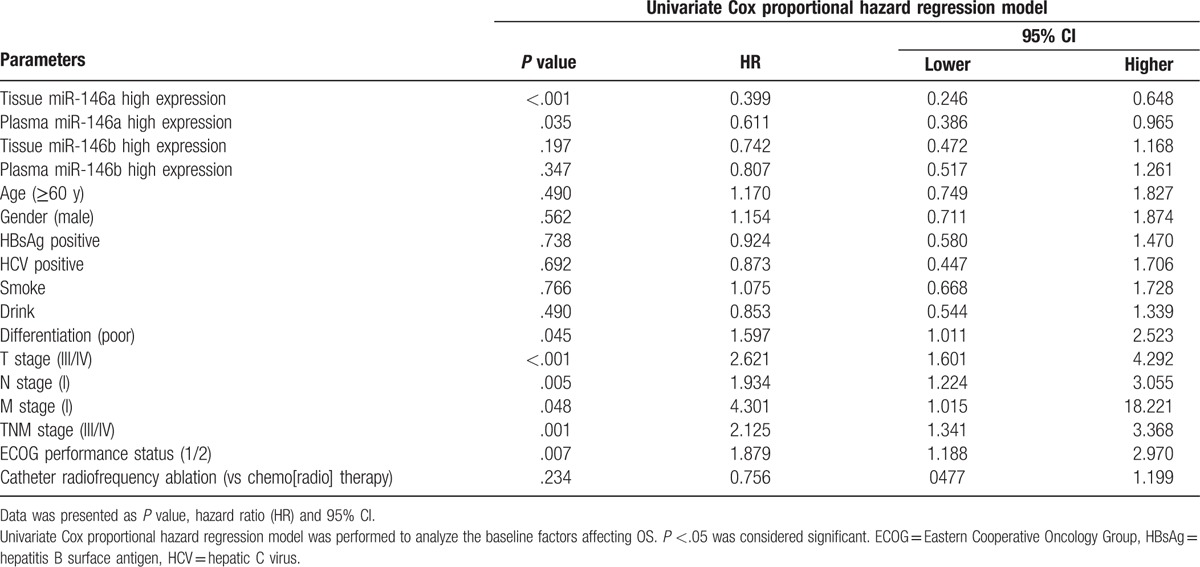
Factors at baseline predicting OS by univariate Cox analysis.

All factors with a *P* value <.1 were further analyzed by the multivariate Cox proportional hazards regression model. It revealed that the high expression of tissue miR-146a (*P* = .001) but not plasma miR-146a (*P* = .076) was an independent factor for prolonged OS, whereas higher T stage (III/IV) (*P* = .002) and evaluated ECOG performance status (1/2) (*P* = .021) could predict worse OS in ICC surgical patients independently (Table 4).

**Table 4:**
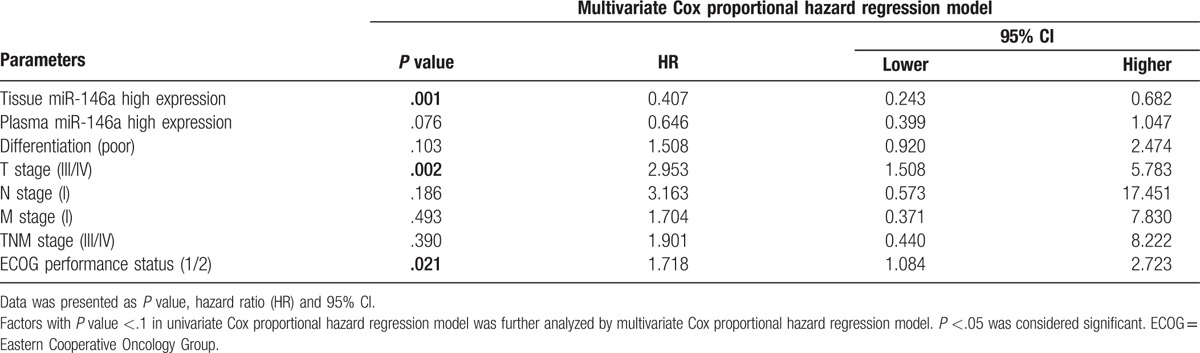
Independent factors at baseline predicting OS by multivariate Cox analysis.

## 4 Discussion

In the present study, we found that both miR-146a and miR-146b expressions in tumor tissue were positively associated with that in plasma; and tumor tissue miR-146a was negatively correlated with age, poor differentiation, N stage, and TNM stage, as well as ECOG performance, whereas plasma miR-146a was inversely associated with N stage, TNM stage, and ECOG performance. As to miR-146b, only tumor tissue was negatively associated with gender and T stage. Both tissue miR-146a and plasma miR-146a were associated with better OS, whereas only tissue miR-146a high expression was an independent factor for prolonged OS of ICC patients, and T stage (III/IV)) and ECOG performance status (1/2) predict worse OS independently.

ICC, one of the multifactorial malignancies, originates from the epithelial cells of the intrahepatic bile duct beyond second-order branches.^[19]^ The most common pathological process of ICC is chronic inflammation and fibrosis of bile duct, which could induce epithelial cells apoptosis and proliferation. Subsequently, this repeated process lead to aberrant molecule activities, stimulating tumorigenesis.^[1]^

miR-146a, encoded on chromosome 5(5q33.3),^[16]^ has been disclosed as a potential tumor repressor in several malignant tumors. Upregulation of miR-146a inhibits lung cancer cell proliferation and differentiation via mediating the macrophage migration inhibitory factor (MIF) gene and NF-κB signaling pathway.^[20]^ Also, its overexpression represses gastric cancer cell migration by reducing the UHRF1 level via targeting its 3′-UTR, resulting in the inhibition of tumor invasion and metastasis subsequently.^[21]^ Additionally, miR-146a expression has been observed to be positively associated with antitumor immune systems by targeting signal transducer and activator of transcription 3 (STAT3) in human hepatocellular carcinoma (HCC) cells.^[22]^ Analysis of miR-146a in triple-negative breast cancer (TNBC) tumors has been observed to predict better OS by regulating breast cancer 1 (BRCA1) expression.^[23]^ As to tissue miR-146, it is considered as a suppressor of HCC, negatively associated with the clinical TNM stage, metastasis, and portal vein tumor embolus, as well as a number of tumor nodes.^[24]^ Furthermore, increased expression of tissue miR-146a has been confirmed to predict a higher survival rate of gastric cancer, esophageal squamous cell carcinoma and non-small cell lung cancer (NSCLC), as well as renal cell carcinoma (RCC).^[25–28]^ These researches suggest that miR-146a acts as tumor suppressor on malignant tumors, and it is correlated with better prognosis. Partially in line with these results, our study found that tumor tissue miR-146a was negatively correlated with poor differentiation, N stage, and TNM stage, as well as ECOG performance, whereas plasma miR-146a was inversely associated with N stage, TNM stage, and ECOG performance. This might arise from the inhibition of cell proliferation, differentiation, and migration by miR-146a through regulating several pathways.^[20,23]^ In addition, we also found that high expressions of tissue miR-146a and plasma miR-146a were associated with better OS, whereas only tissue miR-146a high expression was an independent factor for prolonged OS of ICC patients. There are 2 possible reasons. First, miR-146a inhibits tumor growth and metastasis by mediating various molecule activities^[21,22]^; second, miR-146a increases the sensitivity of chemotherapy, radiotherapy, and targeted therapy, decreasing recurrence of ICC patients.^[29–32]^

miR-146b, consisting of miR-146b-5p and miR-146b-3p, is located on human chromosome 10 at position q24.32.^[33]^ Published studies have defined miR-146b as a tumor inhibitor in various carcinomas, including esophageal cancer, gallbladder cancer and thyroid cancer.^[34–36]^ A larger number of studies have elucidated the role of miR-146b in carcinoma cell lines. In a study of esophageal cancer cell, miR-146b acted as a tumor suppressor by targeting CCAAT/enhancer-binding proteins β liver-enriched transcriptional activator protein 2 (C/EBPβ LAP2) to inducing cell proliferation and repressing cell apoptosis.^[36]^ In addition, miR-146b expression was associated with the TNM stage, liver metastasis and differentiated degree, and its overexpression showed better OS in patients with gallbladder cancer (GBC).^[35]^ These studies illustrates that miR-146b serves as tumor suppressor on the prognosis of carcinomas. On the other hand, miR-146b expression could also act as tumor promoter to inducing thyroid cancer cell migration and invasion by downregulating zinc and ring finger 3 (ZNRF3).^[37]^ However, our study showed that no association of plasma and tissue miR-146b with OS in ICC surgical patients. The possible reasons are that the dual effects of miR-146b on both promotion and repression of malignant tumors. Meanwhile, we also observed that tumor tissue miR-146b was only negatively correlated with T stage, this might result from the repression of cell proliferation and apoptosis via targeting several genes.^[36,38]^

Several limitations still existed in this study. The sample size was relatively small. The plasma miR-146a/b expression of healthy people was not evaluated to compare with ICC patients. The follow-up duration was relatively short with median value 31 months. Further study with larger sample size and longer follow-up duration is necessary to be carried out in the future.

In conclusion, both plasma and tissue miR-146a expression correlated with favorable OS, whereas only tissue miR-146a was an independent prognostic biomarker in surgical patients with ICC.
